# Impact of HIV Infection and Anti-Retroviral Therapy on the Immune Profile of and Microbial Translocation in HIV-Infected Children in Vietnam

**DOI:** 10.3390/ijms17081245

**Published:** 2016-08-02

**Authors:** Xiuqiong Bi, Azumi Ishizaki, Lam Van Nguyen, Kazunori Matsuda, Hung Viet Pham, Chung Thi Thu Phan, Kiyohito Ogata, Thuy Thi Thanh Giang, Thuy Thi Bich Phung, Tuyen Thi Nguyen, Masaharu Tokoro, An Nhat Pham, Dung Thi Khanh Khu, Hiroshi Ichimura

**Affiliations:** 1 Department of Viral Infection and International Health, Graduate School of Medical Sciences, Kanazawa University, Kanazawa 920-8640, Japan; bixiuqio@staff.kanazawa-u.ac.jp (X.B.); azumi0306@aol.com (A.I.); 2 National Hospital of Pediatrics, Hanoi 100-000, Vietnam; dinhlam73@yahoo.com (L.V.N.); vhnhi44@gmail.com (H.V.P.); phanthuchung@gmail.com (C.T.T.P.); huygt2006@gmail.com (T.T.T.G.); phungthuy2707@yahoo.com (T.T.B.P.); ntuyen_nhp@yahoo.com.vn (T.T.N.); nhatan.pham@yahoo.com (A.N.P.); hangdung2001@yahoo.com (D.T.K.K.); 3 Yakult Central Institute, Tokyo 186-8650, Japan; kazunori.matsuda@yher.be (K.M.); ogata@ninesigma.com (K.O.); 4 Department of Parasitology, Graduate School of Medical Sciences, Kanazawa University, Kanazawa 920-8640, Japan; tokoro@med.kanazawa-u.ac.jp

**Keywords:** HIV-infected children, intestinal microbial translocation, immune activation, 16S/23S ribosomal DNA

## Abstract

CD4^+^ T-lymphocyte destruction, microbial translocation, and systemic immune activation are the main mechanisms of the pathogenesis of human immunodeficiency virus type 1 (HIV) infection. To investigate the impact of HIV infection and antiretroviral therapy (ART) on the immune profile of and microbial translocation in HIV-infected children, 60 HIV vertically infected children (31 without ART: HIV(+) and 29 with ART: ART(+)) and 20 HIV-uninfected children (HIV(−)) aged 2–12 years were recruited in Vietnam, and their blood samples were immunologically and bacteriologically analyzed. Among the HIV(+) children, the total CD4^+^-cell and their subset (type 1 helper T-cell (Th1)/Th2/Th17) counts were inversely correlated with age (all *p* < 0.05), whereas regulatory T-cell (Treg) counts and CD4/CD8 ratios had become lower, and the CD38^+^HLA (human leukocyte antigen)-DR^+^CD8^+^- (activated CD8^+^) cell percentage and plasma soluble CD14 (sCD14, a monocyte activation marker) levels had become higher than those of HIV(−) children by the age of 2 years; the CD4/CD8 ratio was inversely correlated with the plasma HIV RNA load and CD8^+^-cell activation status. Among the ART(+) children, the total CD4^+^-cell and Th2/Th17/Treg-subset counts and the CD4/CD8 ratio gradually increased, with estimated ART periods of normalization being 4.8–8.3 years, whereas Th1 counts and the CD8^+^-cell activation status normalized within 1 year of ART initiation. sCD14 levels remained high even after ART initiation. The detection frequency of bacterial 16S/23S ribosomal DNA/RNA in blood did not differ between HIV-infected and -uninfected children. Thus, in children, HIV infection caused a rapid decrease in Treg counts and the early activation of CD8^+^ cells and monocytes, and ART induced rapid Th1 recovery and early CD8^+^-cell activation normalization but had little effect on monocyte activation. The CD4/CD8 ratio could therefore be an additional marker for ART monitoring.

## 1. Introduction

Mucosa-associated lymphoid tissues, such as the gut-associated lymphoid tissue (GALT), harbor approximately 40%–60% of lymphocytes in the human body. GALT is the largest replication site and reservoir of human immunodeficiency virus type 1 (HIV) among HIV-infected individuals. In the early stages of HIV infection, the remarkable destruction of CD4^+^ T (CD4^+^) cells, particularly the type 17 helper T-cell (Th17) subset, occurs in GALT [1,2,3], resulting in the decline in the immune and mechanical barrier functions of the gut mucosa. Subsequently, the translocation of microbial products, such as lipopolysaccharides from gram-negative bacteria and bacterial DNA, from the gastrointestinal tract to systemic circulation occurs, which induces systemic immune activation, disrupts immune balance, and causes further loss of CD4^+^ cells [3,4,5,6]. CD4^+^ cells in the peripheral blood recover 1–3 years after antiretroviral therapy (ART) initiation, whereas the recovery of CD4^+^ cells in GALT is much slower. The barrier function of the gut mucosa therefore remains impaired, and the immune activation continues for many years after ART initiation [3,7,8].

Children who have contracted HIV from their mothers (vertical infection) progress to acquired immunodeficiency syndrome (AIDS) more rapidly than HIV-infected adults, showing a more rapid decline in CD4^+^-cell counts and high plasma HIV viral load (VL). Without ART, more than 50% of these children die by the age of 2 years [9,10]. In children, there is an abundance of lymphoid aggregates in the gut submucosa and memory CD4^+^CCR5^+^ T cells in gut epithelial cells [11]. Because these cells are highly susceptible to HIV infection even without activation, they are likely to be the prime site of HIV infection and replication [9,11]. Furthermore, a relatively low HIV-specific Th1 response coinciding with a low Th1/Th2 ratio and an expanded population of regulatory T cells (Tregs) are observed among HIV-infected children, particularly among infants [9,12,13]. HIV-infected children also exhibit a decrease in CD4^+^-cell counts, CD8^+^-cell activation, and intestinal microbial translocation [14,15], and ART effectively restores CD4^+^-cell populations and suppresses CD8^+^-cell activation [14,16,17], as is the case among HIV-infected adults. However, the impact of HIV infection and ART on different CD4^+^-cell subsets (Th1, Th2, Th17, and Treg) in children remains poorly understood.

Since 2008, we have followed up with HIV-infected children in Hanoi, Vietnam to find an efficient and cost-effective method and immunological markers for monitoring ART in resource-limited settings [18]. Based on the results of that field study, we conducted this cross-sectional study to clarify the impact of HIV infection and ART on the immune profile and microbial translocation status of children aged over 2 years who have an immune status considered to be relatively mature and stable [9,19,20].

## 2. Results

### 2.1. Characteristics of the Subjects

This study included 60 HIV vertically infected children (31 without ART (HIV(+)) and 29 with ART (ART(+)) and 20 HIV-uninfected healthy children (HIV(−)). The characteristics of the study subjects are shown in Table 1. The ART(+) children had received ART for a median period of 3.5 years, and 22 (75.9%) had an undetectable plasma VL (< 220 copies/mL). More ART(+) (5/29, 17.2%) than HIV(+) (1/31, 3.2%) children were at WHO clinical stage 2, although the difference was not statistically significant (*p* = 0.098). The HIV(+) and ART(+) children were 2 years older than the HIV(−) children (HIV(+) vs. HIV(−): median age 6.2 vs. 4.1 years, respectively, *p* = 0.034; ART(+) vs. HIV(−): median age 6.1 vs. 4.1 years, respectively, *p* = 0.009), although height and body weight did not significantly differ among the three groups (all *p* > 0.1).

### 2.2. Immune Status of HIV-Infected Children

The immune statuses of the children in the three groups are shown in Table 1. Compared with the HIV(−) children, the HIV(+) children had significantly lower total CD4^+^-cell (*p* = 0.003) and Th1/Th2/Th17/Treg-subset counts (*p* = 0.003/0.016/<0.001/<0.001, respectively), a lower percentage of CD4^+^ cells in lymphocytes (*p* = 0.001), a higher percentage of CD8^+^ cells in lymphocytes (*p* < 0.001), a lower CD4/CD8 ratio (*p* < 0.001), and a higher percentage of CD38^+^HLA (human leukocyte antigen)-DR^+^CD8^+^ cells in CD8^+^ cells (activated CD8^+^ cells, *p* < 0.001), although CD8^+^-cell counts and the CD4^+^-cell activation status (percentage of CD38^+^HLA-DR^+^CD4^+^ cells in CD4^+^ cells) did not significantly differ between the two groups (*p* = 0.239 and 0.969, respectively). The HIV(+) children had higher plasma soluble CD14 (sCD14; a monocyte activation marker) levels than the HIV(−) children (*p* = 0.009).

The ART(+) children had significantly lower total CD4^+^-cell (*p* = 0.018) and Th2/Th17/Treg-subset counts (*p* = 0.009/<0.001/0.004), a lower CD4/CD8 ratio (*p* = 0.001), and higher sCD14 levels (*p* < 0.001) than the HIV(−) children. However, the CD4^+^-cell percentage in lymphocytes (*p* = 0.404), Th1 count (*p* = 0.611), and activated CD8^+^-cell percentage (*p* = 0.329) did not significantly differ between the two groups.

The ART(+) children had significantly higher Th1//Th17/Treg-subset counts (*p* = 0.002/0.016/<0.001), higher sCD14s levels (*p* < 0.001), and lower activated CD8^+^ percentages (*p* < 0.001) than the HIV(+) children. However, the total CD4^+^-cell (*p* = 0.429) and Th2-subset counts (*p* = 0.970) and the CD4/CD8 ratio (*p* = 0.181) did not significantly differ between the two groups.

### 2.3. Impact of HIV Infection on Immune Profile

**Figure 1:**
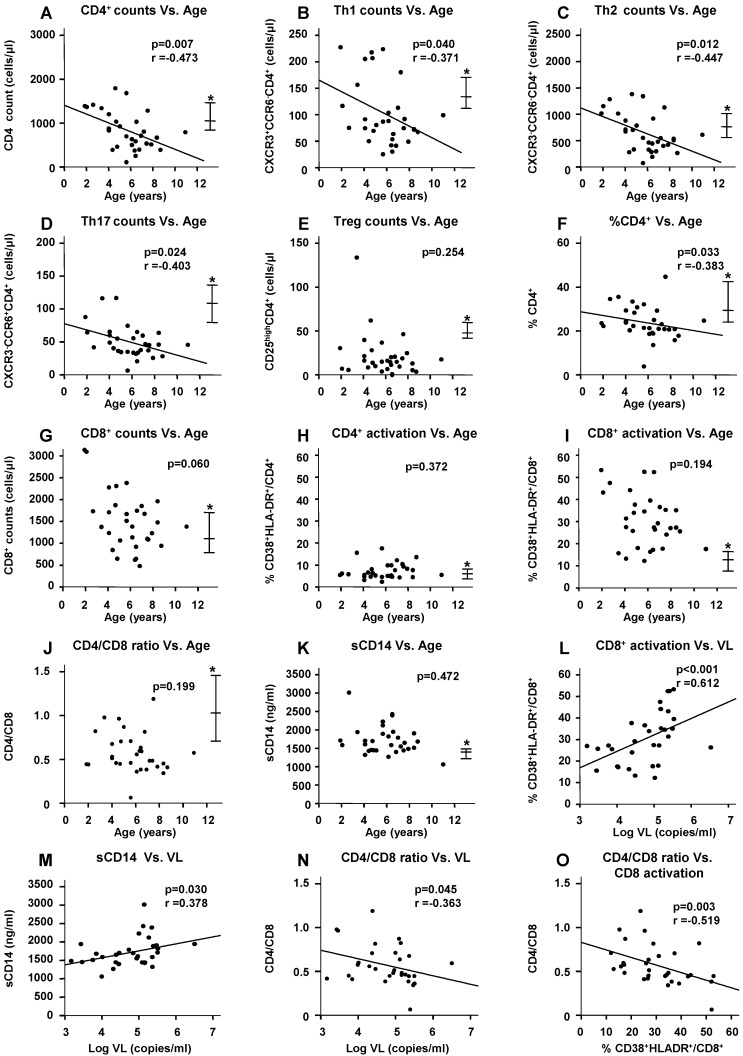
Correlation between immunological markers, age, and/or plasma viral load among HIV(+) children (**A**–**O**). This bivariate correlation was estimated on the basis of Spearman’s rank correlation analysis. Regression lines are shown only for significantly correlated bivariates. Th: helper T-cell, Treg: regulatory T-cell, VL: viral load. * Median and interquartile range of the HIV(−) group.

Among the HIV(+) children, the total CD4^+^-cell counts (Figure 1A, *p* = 0.007), Th1/Th2/Th17-subset counts (Figure 1B–D, *p* = 0.040/0.012/0.024, respectively), CD4^+^-cell percentage in lymphocytes (Figure 1F, *p* = 0.033), and CD8^+^-cell counts (Figure 1G, *p* = 0.060) were inversely correlated with age (that is, nearly equal to their HIV-infection period). On the other hand, Treg counts (Figure 1E) and the CD4/CD8 ratio (Figure 1J) were lower and the CD38^+^HLA-DR^+^CD8^+^- (activated CD8^+^) cell percentage (Figure 1I) and plasma sCD14 levels (Figure 1K) were higher than those of the HIV(−) children by the age of 2 years. The CD38^+^HLA-DR^+^CD4^+^- (activated CD4^+^) cell percentage was not significantly correlated with age (Figure 1H). The activated CD8^+^-cell percentage (Figure 1L, *p* < 0.001) and sCD14 levels (Figure 1M, *p* = 0.030) were positively correlated with the plasma VL. The CD4/CD8 ratio was inversely correlated with the plasma VL (Figure 1N, *p* = 0.045) and CD8 activation status (Figure 1O, *p* = 0.003).

### 2.4. Impact of ART on the HIV-Induced Immune Profile

**Figure 2:**
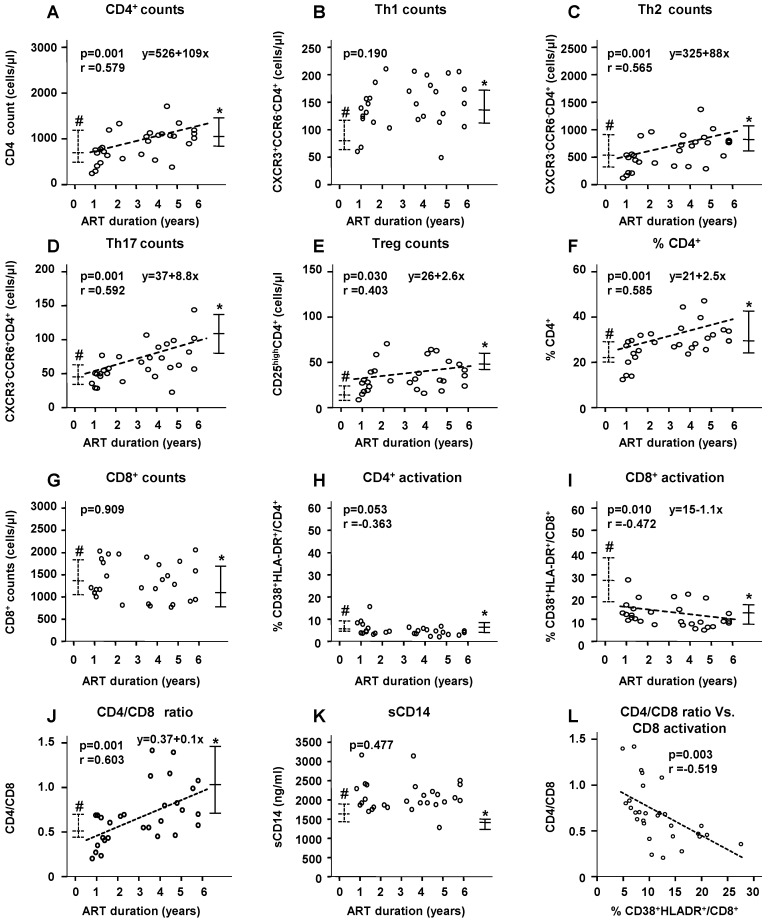
Correlations between ART duration and immunological markers among ART(+) children (**A**–**L**). * Median and interquartile range (IQR) of the HIV(−) group; ^#^ Median and IQR of the HIV(+) group.

Among the ART(+) children, the total CD4^+^-cell counts (Figure 2A, *p* = 0.001), Th2/Th17/Treg-subset counts (Figure 2C–E, *p* = 0.001/0.001/0.030, respectively), CD4^+^-cell percentage in lymphocytes (Figure 2F, *p* = 0.001), and CD4/CD8 ratio (Figure 2J, *p* = 0.001) gradually, but significantly, increased during ART, whereas the Th1 counts had increased to the range of those of the HIV(–) children by around 1 year of ART initiation (Figure 2B). The CD8^+^-cell counts were not significantly correlated with ART duration. None of these markers were significantly correlated with age (data not shown).

Linear regression analyses were conducted to estimate the ART duration required for the immunological markers to reach the median values of the HIV(−) children. The estimations were as follows: 4.8 years (95% confidence interval (CI): 2.7–7.0 years) of ART for the total CD4^+^-cell counts, 3.6 years (95% CI: 1.4–5.7) for the CD4^+^-cell percentage in lymphocytes, 5.6 years (95% CI: 3.4–7.9) for the Th2 counts, 8.2 years (95% CI: 6.2–10.2) for the Th17 counts, 8.3 years (95% CI: 3.4–12.2) for the Treg counts, and 6.6 years (95% CI: 4.5–8.7) for the CD4/CD8 ratio. 

During the first year of ART initiation, the percentage of activated CD8^+^ cells rapidly decreased to the upper range of that among the HIV(–) children and then continued to slowly, but significantly, decrease (Figure 2I, *p* = 0.010). In contrast, the percentage of activated CD4^+^ cells marginally decreased with ART duration (Figure 2H, *p* = 0.053), but the percentage of activated CD4^+^ cells even among the HIV(+) children did not significantly differ from that among the HIV(−) children (5.6% vs. 6.4%, *p* = 0.969, Table 1). The plasma sCD14 levels showed no significant changes during almost 6 years of ART (Figure 2K).

The CD4/CD8 ratio was inversely correlated with the percentage of activated CD8^+^ cells (*p* = 0.003, Figure 2L).

### 2.5. Physiological Change in Immunological Markers with Age among the HIV(−) Children

**Figure 3:**
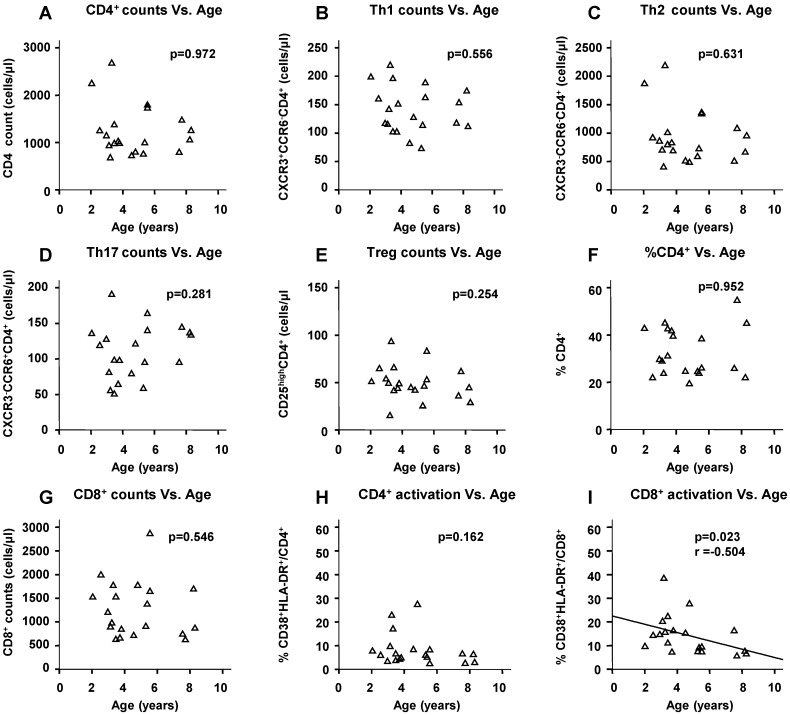

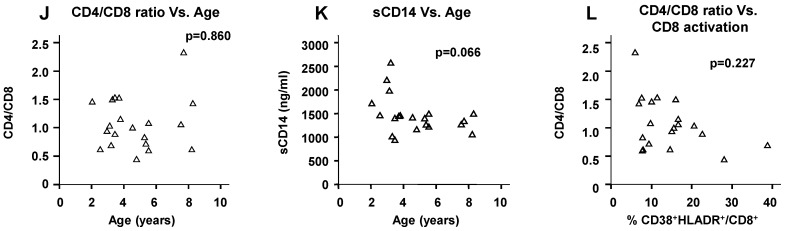
Correlation between immunological markers and age among HIV(−) children (**A**–**L**). This bivariate correlation was estimated on the basis of Spearman’s rank correlation analysis. Regression lines are shown only for significantly correlated bivariates.

Among the HIV(−) children, only the percentage of activated CD8^+^ cells significantly decreased with age (*p* = 0.023, Figure 3I), and plasma sCD14 levels marginally decreased with time by 4 years of age and remained stable thereafter (*p* = 0.066, Figure 3K). The other immunological markers did not show significant relationships with age (Figure 3A–H,J,L).

### 2.6. Microbial Translocation Status

The impact of HIV infection and ART on the microbial translocation status of the children was determined by the detection of 12 bacterial 16S/23S ribosomal RNA genes (rDNA) in plasma and 16S/23S ribosomal RNA molecules (rRNA) in whole blood using quantitative PCR (qPCR) and reverse transcription (RT)-qPCR, respectively. Bacterial 16S/23S rDNA from *Staphylococcus*, *Streptococcus*, and/or *Pseudomonas* species was detected in 25.8% (8/31) of the HIV(+) children compared with 15.0% (3/20) of the HIV(−) children (Table 2). The detection frequency of *Staphylococcus* rDNA was significantly higher among the HIV(+) children than among the ART(+) children (22.6% vs. 0%, respectively, *p* = 0.011), whereas there was no significant difference between the HIV(+) and HIV(−) children in terms of the frequency of each bacterial rDNA (*p* = 0.169) and the number of bacterial DNA copies (data not shown).

No target bacterial 16S/23S rRNA was detected in children, regardless of the HIV status (data not shown).

## 3. Discussion

In the current study, we investigated the impact of HIV infection and ART on the immune profile of children aged over 2 years. Among the HIV(+) children, the total counts of CD4^+^ cells and their subsets (Th1/Th2/Th17) were inversely correlated with age, whereas Treg counts, CD38^+^HLA (human leukocyte antigen)-DR^+^CD8^+^- (activated CD8^+^) cell percentage, and plasma sCD14 levels (activated monocyte level) were not. By the age of 2 years, Treg counts had become lower and the activated CD8^+^ cell and monocyte levels had become higher than those of the HIV(−) children. In addition, activated CD8^+^ cell and monocyte levels were positively correlated with VL, and the CD8^+^-cell activation status was inversely, although marginally, correlated with Treg counts, (*p* = 0.0575). These findings indicate that vertical HIV infection induces a rapid decrease in the Treg subset and the early activation of CD8^+^ cells and monocytes. These data suggest that the early decline in Tregs contributes to CD8^+^-cell activation in the early phase of HIV infection in children, as previously reported in adults [21]. The early activation of CD8^+^ cells after HIV infection [15,16] and a correlation between CD8^+^ cell activation and VL [22] have been previously reported, whereas a rapid decrease in Treg subset has not been previously reported in children, although it has been reported in adults with primary HIV infection [23].

Among the ART(+) children, Th2/Th17/Treg-subset counts were positively correlated with ART duration and were estimated to reach the levels of HIV(−) children after 5.6, 8.2, and 8.3 years of ART initiation, respectively, compared with an estimate of only 1–2 years for Th1 counts. The activated CD8^+^ cell percentage decreased to the level of the HIV(−) children during the first year of ART initiation, whereas the plasma sCD14 level (activated monocytes level) was maintained at a level higher than that of the HIV(−) children even after VL was controlled and CD8^+^-cell activation was normalized. These findings indicate that in children, ART induced a more rapid Th1 recovery than the Th2/Th17/Treg subsets and a rapid normalization of CD8^+^-cell activation but had little effect on monocyte activation. Although previous studies have reported the early normalization of CD8^+^-cell activation after ART initiation [16,24], the recovery profile of CD4^+^-cell subsets after ART initiation, particularly the rapid recovery of Th1 and the slower recovery of Th2/Th17/Treg, has not been reported before in children. Incidentally, the rapid recovery of Th1 may play a role in the control of HIV infection together with ART. Similar to our findings, a previous study has reported that the plasma sCD14 level is not normalized even after 2 years of ART initiation in children, although it is significantly reduced after ART initiation [25]. Because the sCD14 level has been proposed as an independent predictor of non-AIDS-defining morbidity events even during suppressive ART in HIV-infected adults [26,27], further longitudinal studies may be needed to elucidate the implication of the high monocyte activation status in HIV-infected children under ART.

Among the HIV(+) children, the CD4/CD8 ratio was inversely correlated with the plasma VL and the activated CD8^+^-cell percentage. Among the ART(+) children, the CD4/CD8 ratio was positively correlated with ART duration, as were the total CD4^+^-cell and Th2/Th17/Treg-subset counts, and inversely correlated with the percentage of activated CD8^+^ cells. Recent studies have suggested that the CD4/CD8 ratio is a marker of T-cell activation, senescence, and activation/exhaustion in treated HIV-infected children and young adults, and that it could be independently associated with the risk of non-AIDS-related morbidity and mortality [28,29]. Given the cost effectiveness of measuring CD4^+^ and CD8^+^ cell levels in clinical laboratories, the CD4/CD8 ratio could be used as an additional immunological marker to monitor ART outcomes, particularly in resource-limited settings.

Intestinal microbial translocation plays a role in the pathogenesis of HIV infection [6]. Furthermore, immune activation is driven by intestinal microbial translocation in HIV-infected children both before and after ART [22,30]. However, in this study, none of the targeted bacterial 16S/23S rRNA fragments were detected in whole blood samples of the 80 study participants, indicating that live bacteria (bacteremia) are rarely detected in HIV-infected and -uninfected children. In contrast, bacterial rDNA was detected in 8 HIV(+) children and 3 HIV(−) children, and the detection frequency of staphylococcal rDNA was significantly higher among the HIV(+) children than among the ART(+) children (22.6% vs. 0%, respectively). However, there was no significant association of the copy number of bacterial 16S/23S rDNA with CD8^+^-cell or monocyte activation and no significant difference in the frequency of bacterial rDNA between the HIV(+) and HIV(−) children. These data suggest that ART decreases the occurrence of intestinal microbial translocation in HIV-infected children, although the possibility of contamination with *Staphylococcus epidermidis* cannot be excluded. Thus, further studies are needed to elucidate the reasons for the discrepancies between our data and previous findings [22,30] in relation to microbial translocation in HIV-infected children.

In this study, we recruited children who were over 2 years of age because their immune systems are considered to be relatively mature and stable [9,19,20]. We showed that all targeted immunological markers, except CD8^+^-cell activation, did not significantly change with age among the HIV(–) children (Figure 3). These data demonstrate that the results presented here on the impact of HIV infection and ART on the immune profile of children were not influenced by growth-related physiological changes.

There are some limitations to this study. First, this was a cross-sectional study rather than a longitudinal study; Second, the number of study subjects was relatively small, which limited our findings in relation to microbial translocation; Third, the HIV(+) and ART(+) children were 2 years older than the HIV(−) children; Finally, only cell-surface markers were used to identify the CD4^+^-subsets, particularly Treg. Therefore, these findings should be confirmed by longitudinal studies following HIV-infected children before and during ART with age-matched controls.

To our knowledge, this is the first study to investigate the impact of HIV infection and ART on the immune profile of CD4^+^-cell subsets (Th1/Th2/Th17/Tregs) concurrently with the activation of T cells and monocytes in children. We found that HIV infection induced a more rapid decline in the Treg subset than in the Th1/Th2/Th17 subsets and the early activation of CD8^+^ cells and monocytes. We also found that ART induced a more rapid recovery of the Th1 subset than the Th2/Th17/Treg subsets and the early normalization of CD8^+^-cell activation but that it had little effect on the activation of monocytes in children. Finally, we have provided evidence that the CD4/CD8 ratio is an additional marker for ART outcomes.

## 4. Materials and Methods

### 4.1. Subjects and Study Design

Sixty Vietnamese children vertically infected with HIV were recruited in May 2012. These children were assigned to one of two groups: the HIV(+) group and the ART(+) group. The HIV(+) group comprised 31 children with HIV who did not receive ART; the female/male ratio was 14/17, and the median age was 6.2 years (2.0–11.0 years). The ART(+) group comprised 29 children with HIV who had been treated with ART; the female/male ratio was 12/17, and the median age was 6.1 years (3.6–8.6 years). The study inclusion criteria for these HIV-1-infected groups were as follows: the children (1) had been followed at the National Hospital of Pediatrics (NHP) in Hanoi, Vietnam; and (2) were more than 2 years of age because at this point the immune system is relatively mature and stable [9,19,20]. The exclusion criteria were as follows: children who (1) had progressed to AIDS; (2) had received any treatment within the prior 8 weeks that might influence the immune system; and (3) had symptoms of gastrointestinal infections at the time of recruitment. The children in the ART(+) group resided at an orphanage center near Hanoi; the children in the HIV(+) group were followed at the outpatient department of NHP. A third control group, the HIV(−) group, was also included and comprised 20 healthy Vietnamese children without HIV infections. The female/male ratio was 8/12, and the median age was 4.1 years (2.0–8.3 years). The children in this group resided at an orphanage center near Hanoi. The characteristics of the study subjects are shown in Table 1.

At recruitment, the median ART duration in the ART(+) group was 3.5 years (0.8–5.8) years. Of the 29 children, 8 received zidovudine (AZT)/lamivudine (3TC)/nevirapine (NVP), 7 received stavudine (d4T)/3TC/NVP, 6 received AZT/3TC/efavirenz (EFV), 4 received d4T/3TC/EFV, 2 received AZT/3TC/lopinavir boosted with ritonavir (LPV/r), 1 received abacavir (ABC)/3TC/LPV/r, and 1 received ABC/didanosine/LPV/r.

The protocol of this cross-sectional study was approved by the Ethics Committee of Kanazawa University in Japan and the Ethics Committee of NHP in Vietnam. All of the HIV-infected children who met the inclusion criteria and did not correspond to the exclusion criteria, and all of the HIV-uninfected children who resided in the orphanage and were eligible for the criteria, were invited to join this study. The family or guardian of each subject was informed, and only those who voluntarily consented to participate were recruited. Written consent was obtained from all participants.

### 4.2. Plasma HIV VL and sCD14 Concentration

Plasma HIV VL was measured using a Cobas Taqman HIV-1 Test Kit version 1.0 (Roche Molecular Systems, Inc., Branchburg, NJ, USA), following the manufacturer’s instructions (detection limit: 40 copies/mL). Plasma samples were diluted to 1:5.5 for measurements, which resulted in a final detection limit of 220 copies/mL.

Plasma sCD14 concentration was measured with a Human sCD14 Immunoassay Kit (R&D Systems, Minneapolis, MN, USA), following the manufacturer’s instructions.

### 4.3. Immunological Analysis

Immune activation was evaluated based on the percentage of CD4^+^ and CD8^+^ lymphocytes expressing CD38 and major histocompatibility complex class II (HLA-DR) molecules [31]. The different CD4^+^-cell subsets were identified using cell-surface markers: CXCR3^+^CCR6^−^CD4^+^ (“Th1”), CXCR3^−^CCR6^−^CD4^+^ (“Th2”), CXCR3^−^CCR6^+^CD4^+^ (“Th17”) [32], and CD25^high^CD4^+^ (“Treg”) [33].

Blood samples were processed for cell staining within 6 h after collection. Whole blood samples (50 µL) were stained for 15 min at 4 °C with a combination of four monoclonal antibodies: anti-CD4 PerCP and CD8 PE (BD Biosciences, San Jose, CA, USA), CD38 FITC (Miltenyi Biotec, Auburn, AL, USA), and HLA-DR PE-Cy7 (Biolegend, San Diego, CA, USA). Alternatively, samples were stained with a combination of four anti-CD4 monoclonal antibodies: anti-CD4 PerCP and CD25 PE (BD Biosciences), and CXCR3 FITC and CCR6 PE-Cy7 (Biolegend). After red blood cells were lysed with a lysing buffer (BD Biosciences), the remaining cells were washed once with 1 mL of phosphate buffered saline (PBS), kept at 4 °C in PBS with 1% paraformaldehyde and 0.5% bovine serum albumin, and analyzed within 48 h after staining at Kanazawa University with a JSAN flow cytometer (Bay Bioscience, Kobe, Japan). The data were analyzed with Flowjo V.7.5.5 (FLOWJO, OR, USA).

### 4.4. Detection of Bacterial Ribosomal RNA Genes (rDNA) in Plasma

Isolated plasma (100 µL) was used for DNA extraction with the SMI TEST EX R&D (Medical and Biological Laboratories Co., Ltd., Aichi, Japan), following the manufacturer’s instructions. qPCR was then performed with a TaKaRa Taq kit (TaKaRa Bio Inc., Shiga, Japan). The reaction mixture (10 μL) contained 5 μL of template DNA and 0.2 μM of each specific primer set, except for the primer targeting g-Bfra-F2/g-Bfra-R (0.4 μM). The primers were designed to target bacterial 16S or 23S rRNA genes (Table S1). The amplification program comprised one cycle at 94 °C for 5 min and 45 cycles at 94 °C for 20 s, 55 °C or 60 °C for 20 s, and 72 °C for 50 s. A standard curve was generated with qPCR data and cycle threshold (*C*_t_) values. The target gene copy number in the plasma samples was determined in a sample of the extracted DNA (1/20 of the DNA extracted from 100 µL of plasma). This DNA was subjected to qPCR, and the *C*_t_ value was applied to the standard curve to obtain the corresponding bacterial rDNA copy number/µL of plasma. With this procedure, the lower detection limit for the targeted gene was 2 copies/µL of plasma.

### 4.5. Detection of Bacterial rRNA in Blood

Peripheral blood (1 mL) was added to two volumes of the RNA Protect bacterial reagent (QIAGEN GmbH, Hilden, Germany). After centrifugation of the mixture at 14,000× *g* for 10 min, the supernatant was discarded and the pellet was stored at −80 °C until further use. Total RNA extraction was followed by RT-qPCR as previously described [34]. Each RNA sample was diluted; then, diluted samples (corresponding to amounts of 1/200 and 1/20 of the extracted RNA from 1 mL of blood) were subjected to RT-qPCR with specific primer sets that targeted bacterial 16S/23S rRNA [34,35] to investigate if live bacteria existed in the blood stream of the study subjects (Table S1). With this procedure, the lower detection limit for the targeted bacteria was 4 cells/mL of blood.

### 4.6. Statistical Analysis

Statistical analyses were performed with the SPSS programs (IBMSPSS statistics 19, IBM Corporation, NY, USA). The Mann–Whitney *U* test was used to compare the markers among the three groups (HIV(−), HIV(+),and ART(+)). Fisher’s exact test or the chi square test was used to compare the detection frequency of bacterial rDNA in plasma, sex distribution, and the WHO clinical stage across the groups. Spearman’s rank correlation was used to analyze the correlation among the biological markers in each group. *p*-values < 0.05 were considered statistically significant.

## 5. Conclusions

In children, HIV infection caused a rapid decrease in Treg counts and the early activation of CD8^+^ cells and monocytes, and ART induced rapid Th1 recovery and early CD8^+^-cell activation normalization but had little effect on monocyte activation. The CD4/CD8 ratio could be an additional marker for ART monitoring.

## Figures and Tables

**Table 1:** Characteristics and immune status of each group.

Items	HIV(+) (*n* = 31)	HIV(−) (*n* = 20)	ART(+) (*n* = 29)	*p* Values		
				HIV(+) vs. HIV(−)	ART(+) vs. HIV(−)	HIV(+) vs. ART(+)
Age (years) *	6.2 (2.0–11.0)	4.1 (2.0–8.3)	6.1 (3.6–8.6)	0.034	0.009	0.584
Gender, female (*n*)/male (*n*) *	14/17	8/12	12/17	0.718	0.920	0.764
Height (cm) *	109.0 (77.0–129.5)	110.0 (80.0–130.0)	110.0 (90.0–130.0)	0.771	0.418	0.539
Body weight (kg) *	17.5 (10.0–27.0)	16.0 (9.0–35.0)	19.8 (12.0–32.8)	0.395	0.140	0.192
WHO clinical stage: 2 (*n*)/1 (*n*)	1/30		5/24	-	-	0.098
ART duration (years) *			3.5 (0.8–5.8)	-	-	-
Viral load (log_10_ copies/mL) *	5.0 (3.2–6.5)		**	-	-	< 0.001
% CD4^+^ *	22.1 (3.6–44.5)	29.5 (19.6–54.9)	28.8 (12.4–47.1)	0.001	0.404	0.010
CD4^+^-cell counts (cells/μL) *	698 (97–1784)	1050 (693–2688)	894 (244–1711)	0.003	0.018	0.429
Th1 counts (cells/μL) *	80 (25–227)	136 (74–220)	147 (49–211)	0.003	0.611	0.002
Th2 counts (cells/μL) *	537 (63–1375)	822 (413–2196)	553 (119–1369)	0.016	0.009	0.970
Th17 counts (cells/μL) *	45 (6–116)	109 (51–192)	58 (23–144)	<0.001	<0.001	0.016
Treg counts (cells/μL) *	14 (0–133)	48 (16–94)	30 (9–71)	<0.001	0.004	<0.001
%CD38^+^HLA-DR^+^/CD4 *	5.6 (2.2–17.3)	6.4 (2.7–27.6)	4.3 (2.0–15.6)	0.969	0.036	0.003
% CD8^+^ *	43.4 (29.5–61.4)	31.4 (23.5–43.3)	44.7 (31.1–61.4)	<0.001	<0.001	0.464
CD8^+^-cell counts (cells/μL) *	1368 (470–3127)	1101 (634–2874)	1212 (769–2064)	0.239	0.290	0.631
%CD38^+^HLA-DR^+^/CD8 *	27.5 (12.2–53.3)	12.9 (5.8–38.6)	10.2 (5.0–27.7)	<0.001	0.329	<0.001
CD4/CD8 *	0.50 (0.06–1.19)	1.03 (0.45–2.34)	0.66 (0.20–1.42)	<0.001	0.001	0.181
sCD14 (ng/mL) *	1637 (1049–3003)	1413 (944–2580)	1964 (1281–3169)	0.009	<0.001	<0.001

HIV(+): Children infected with HIV and without ART; ART(+): Children infected with HIV and on ART; HIV(−): Children not infected with HIV. P values are from the Man-Whitney *U* test, except the *p* values for sex and WHO clinical stage comparison, which are from the chi square test or Fisher’s exact test. * Median (range); ** 22 ART(+) children with undetectable viral load.

**Table 2:** Bacterial 16S/23S ribosomal RNA gene (rDNA) detection in plasma.

Target Bacteria	HIV(+) (*n* = 31)	HIV(−) (*n* = 20)	ART(+) (*n* = 29)	*p* Values		
				HIV(+) vs. HIV(−)	ART(+) vs. HIV(−)	HIV(+) vs. ART(+)
*C. coccoides* group	0	0	0	-	-	-
*C. leptum* subgroup	0	0	0	-	-	-
*B. fragilis* group	0	0	0	-	-	-
*Bifidobacterium*	0	0	0	-	-	-
*Atopobium* cluster	0	0	0	-	-	-
*Prevotella*	0	0	0	-	-	-
*Enterobacteriaceae*	0	0	0	-	-	-
*Streptococcus*	1 (3.2%)	0	0	1	-	1
*Enterococcus*	0	0	0	-	-	-
*Staphylococcus*	7 (22.6%)	1 (5.0%)	0	0.13	0.41	0.011
*Pseudomonas*	1 (3.2%)	2 (10.0%)	0	0.55	0.16	1
*L. casei* subgroup	0	0	0	-	-	-

HIV(+): Children infected with HIV and without ART; ART(+): Children infected with HIV and on ART; HIV(−): Children not infected with HIV; *C. coccoide*: *Clostridium coccoide*; *C. leptum*; *Clostridium leptum*; *B. fragilis*: *Bacteroides fragilis*. *p* values: Fisher’s exact probability test.

## Supplementary Materials

Supplementary materials can be found at http://www.mdpi.com/1422-0067/17/8/1245/s1.
