# Supplementary material for: Impact of HIV Infection and Anti-Retroviral Therapy on the Immune Profile of and Microbial Translocation in HIV-Infected Children in Vietnam
Source: Int J Mol Sci. 2016 Aug 2;17(8):1245. doi: 10.3390/ijms17081245 (PMC5000643; doi:10.3390/ijms17081245)
Supplement: Supplementary file 1 [file ijms-17-01245-s001.pdf]

Supplementary Materials: Impact of HIV Infection and Anti-Retroviral Therapy on the Immune Profile of and Microbial Translocation in HIV-Infected Children in Vietnam

Xiuqiong Bi, Azumi Ishizaki, Lam Van Nguyen, Kazunori Matsuda, Hung Viet Pham, Chung Thi Thu Phan, Kiyohito Ogata, Thuy Thi Thanh Giang, Thuy Thi Bich Phung, Tuyen Thi Nguyen, Masaharu Tokoro, An Nhat Pham, Dung Thi Khanh Khu and Hiroshi Ichimura

Table S1. Primers used for the target bacterial 16S/23S rRNA/DNA genes.

Target Bacteria	Primer	Sequence (5′–3′)
Clostridium coccoides group	g-Ccoc-F	AAATGACGGTACCTGACTAA
	g-Ccoc-R	CTTTGAGTTTCATTCTTGCGAA
Clostridium leptum subgroup	sg-Clept-F	GCACAAGCAGTGGAGT
	sg-Clept-R3	CTTCCTCCGTTTTGTCAA
Bacteroides fragilis group	g-Bfra-F2	AYAGCCTTTCGAAAGRAAGAT
	g-Bfra-R	CCAGTATCAACTGCAATTTTA
Bifidobacterium	g-Bifid-F	CTCCTGGAAACGGGTGG
	g-Bifid-R	GGTGTTCTTCCCGATATCTACA
Atopobium cluster	c-Atopo-F	GGGTTGAGAGACCGACC
	c-Atopo-R	CGGRGCTTCTTCTGCAGG
Prevotella	g-Prevo-F	CACRGTAAACGATGGATGCC
	g-Prevo-R	GGTCGGGTTGCAGACC
Enterobacteriaceae	En-lsu-3F	TGCCGTAACTTCGGGAGAAGGCA
	En-lsu-3'R	TCAAGGACCAGTGTTCAGTGTC
Lactobacillus casei subgroup	sg-Lcas-F	ACCGCATGGTTCTTGGC
	sg-Lcas-R	CCGACAACAGTTACTCTGCC
Streptococcus	g-Str-F	AGCTTAGAAGCAGCTATTCATTC
	g-Str-R	GGATACACCTTTCGGTCTCTC
Enterococcus	g-Encoc-F	ATCAGAGGGGGATAACACTT
	g-Encoc-R	ACTCTCATCCTTGTTCTTCTC
Staphylococcus	g-Staph-F	TTTGGGCTACACACGTGCTACAATGGACAA
	g-Staph-R	AACAACTTTATGGGATTTGCWTGA
Pseudomonas	PSD7F	CAAAACTACTGAGCTAGAGTACG
	PSD7R	TAAGATCTCAAGGATCCCAACGGCT
